# Complete Genome Sequences of Five Human Respiratory Syncytial Virus Isolates Collected in Brazil

**DOI:** 10.1128/genomeA.01609-17

**Published:** 2018-02-15

**Authors:** Nicholas Di Paola, Marielton dos Passos Cunha, Giuliana Stravinskas Durigon, Eitan Naaman Berezin, Edison Durigon, Danielle Bruna Leal de Oliveira, Paolo Marinho de Andrade Zanotto

**Affiliations:** a Department of Microbiology, Institute of Biomedical Sciences, University of São Paulo, São Paulo, Brazil; b Department of Pediatrics, Santa Casa de Misericórdia Hospital, São Paulo, Brazil; c Infectious Disease Division, Children’s Institute University of São Paulo, HC-FMUSP, São Paulo, Brazil

## Abstract

Here, we present the complete genome sequences of five human respiratory syncytial virus isolates collected from hospitalized infants suffering from acute respiratory disease. These are the first five complete genome sequences of human respiratory syncytial virus to originate from Brazil.

## GENOME ANNOUNCEMENT

Human respiratory syncytial virus (HRSV) is a negative-sense single-stranded RNA virus and is part of the *Orthopneumovirus* genus within the *Pneumoviridae* family. HRSV is a causative agent of severe lower respiratory tract infection in infants and children (1). Nearly all children over the age of 2 years have been infected by HRSV (2). In Brazil, HRSV was detected in 41.8% of patients under 2 years of age with a lower respiratory tract infection (3).

Patients under 2 years of age were included in a prospective study of acute respiratory infection surveillance. Patients with signs, symptoms, and/or a history of lower respiratory tract infections at the time of their admission were included in this study. Specifically, patients with signs or symptoms of wheezing, whooping cough, pertussis-like syndrome, croup, cyanosis, alveolar pneumonia, apnea, bronchiolitis, and bronchospasms were considered. Nasopharyngeal aspirates from patients were first subjected to all PCR diagnostic assays for common respiratory viruses; all were found to be negative. To discover the etiological pathogen and improve surveillance, patient samples were processed for next-generation sequencing.

Viral RNA was extracted from the nasopharyngeal aspirates using the QIAamp viral RNA minikit (Qiagen, Valencia, CA, USA), purified with DNase I, and concentrated using the RNA Clean & Concentrator TM-5 kit (Zymo Research, Irvine, CA, USA). The paired-end RNA libraries were constructed and validated using the TruSeq stranded total RNA high-throughput (HT) sample prep kit (Illumina, San Diego, CA, USA). Sequencing was done at the Core Facility for Scientific Research–University of São Paulo (CEFAP-USP/GENIAL) using the Illumina NextSeq platform. Each sample was barcoded individually, which allowed separation of reads for each patient. Short unpaired reads and bases and low-quality reads were removed using Trimmomatic version 0.36 (4). Paired-end reads (Phred quality score, >33) were assembled *de novo* with SPAdes version 3.10 using default parameters (5).

The *de novo* assemblies of five isolates constructed contigs ranging from 15,181 to 15,268 nucleotides (nt) in length. The average depths of the assemblies ranged from 12× to 475×. Using Geneious 9.1.2, we extracted the consensus sequences (6) and used BLASTn to identify HRSV and class the subtype of each HRSV isolate. STA754, STA786, and STA826 were most closely related to HRSV subtype B (HRSV-B) isolates, while STA836 and STA839 were more related to HRSV subtype A (HRSV-A). When our HRSV-A isolates were compared to the HRSV-A reference genome sequence (GenBank accession number NC_001803), pairwise identities were 97% (STA836) and 96% (STA839). Similarly, for HRSV-B isolates, pairwise identities were also 97% (STA754) and 96% (STA786 and STA826) compared to the HRSV-B reference genome sequence (GenBank accession number NC_001781).

The reported sequences are the first complete HRSV genome sequences from Brazil.

### Accession number(s).

The complete genome sequences of the STA786 (HRSV-B), STA826 (HRSV-B), STA754 (HRSV-B), STA836 (HRSV-A), and STA839 (HRSV-A) isolates have been submitted to GenBank under the accession numbers MG431251 to MG431255, respectively.
